# A Sensitive Monoclonal-Antibody-Based ELISA for Forchlorfenuron Residue Analysis in Food Samples

**DOI:** 10.3390/bios12020078

**Published:** 2022-01-28

**Authors:** Xinmei Liu, Bo Xie, Yongjian Cheng, Lin Luo, Yifan Liang, Zhili Xiao

**Affiliations:** Guangdong Provincial Key Laboratory of Food Quality and Safety, College of Food Science, South China Agricultural University, Guangzhou 510642, China; liuxinmei312@163.com (X.L.); xiebo950216@163.com (B.X.); 18815593698@163.com (Y.C.); lin.luo@scau.edu.cn (L.L.); yfliang0605@163.com (Y.L.)

**Keywords:** forchlorfenuron, monoclonal antibody, icELISA

## Abstract

In this study, forchlorfenuron (CPPU) was coupled with succinic anhydride to yield a CPPU hapten (CPPU-COOH), and a high-affinity monoclonal antibody (mAb) that can specifically recognize CPPU was produced. Using this mAb as a recognition reagent, a sensitive indirect competitive enzyme-linked immunosorbent assay (icELISA) for CPPU was optimized, which exhibits an IC_50_ of 1.04 ng/mL, a limit of detection of 0.16 ng/mL, and a linear range of 0.31–3.43 ng/mL for CPPU. Cross-reactivity percentages with six analogues were all below 6%. The average recovery rates for cucumber and orange samples were from 85.23% to 119.14%. The analysis results of this icELISA showed good consistency with those from liquid chromatography mass spectrometry. These results suggest that the proposed icELISA provides a sensitive, specific, and reliable strategy for CPPU detection in food samples.

## 1. Introduction

Forchlorfenuron (CPPU) is a widely used plant growth regulator that can boost the yields of vegetable and fruit crops [1,2]. Several studies have reported that excessive intake of CPPU can potentially damage human health [3,4,5]. Zhu et al. [6] found that CPPU exposure can stimulate estradiol secretion, resulting in an earlier time of vaginal opening and first estrus time in prepubertal female rats. Gong et al. [7,8] found that CPPU can induce cardiac morphology deformation and validated the cardiotoxicity of CPPU in a different experimental model. A chronic toxicity study on CPPU revealed its potentially negative effects on the ovaries [9]. In addition, long-term exposure to CPPU can induce acute and chronic intoxication and irritation to the eyes and skin [10]. Hence, to protect public health, many countries have issued a maximum residue limit (MRL) for CPPU. The MRL for CPPU in grapes and kiwifruit is 0.05 mg/kg in China, and 0.01 mg/kg in the European Union (EU). For cucumber and orange crops, the MRL is 0.1 mg/kg and 0.05 mg/kg, respectively, in China, and 0.01 mg/kg for both crops in the EU [11,12].

Many techniques have been developed for the purpose of analyzing CPPU residue levels in food samples, such as Raman spectroscopy [3,13] and high-performance liquid chromatography (HPLC) [4,14,15,16]. Most of these techniques can achieve the sensitive and accurate quantification of CPPU; however, they are quite time-consuming and require expensive instruments operated by professional personnel. In comparison, immunoassays (IAs) possess the merit of being cost-effective; they use high-throughput screening, have high specificity, and simple sample preparation. They have become a favorable complement to the use of instrumental methods when screening large numbers of samples [17,18].

Up to now, several IAs for the detection of CPPU have already been developed. Abad-Fuentes’ group [19,20,21] synthesized a set of haptens against CPPU by introducing linear aliphatic spacers at different sites of the phenyl ring or the pyridyl ring in CPPU; all the haptens were able to induce high-affinity antisera to CPPU (IC_50_ < 1 nM). A monoclonal-antibody-based enzyme-linked immunosorbent assay (ELISA) for CPPU detection was developed with a limit of quantification of 5 μg/kg for kiwifruit, and a remarkable cross-reactivity (CR) of 71% with the herbicide thidiazuron (TDZ) was observed. Lu et al. [22] synthesized an immunogen and a coating antigen (CPPU-H1-BSA, CPPU-H2-OVA) by referring to the strategy of Abad-Fuentes [21] and established a monoclonal antibody (mAb)-based indirect competitive ELISA (icELISA) for CPPU. The IC_50_ was 3.89 ng/mL. Compared with the hapten synthetic routes of Abad-Fuentes, which involve three to five steps of reactions, Xiao [23] reported a CPPU hapten synthesized through a more convenient strategy. The CPPU was coupled with succinic anhydride via a one-step Friedel–Crafts reaction, and a spacer arm was introduced on the phenyl ring of the CPPU. The hapten was conjugated with carrier proteins to obtain artificial antigens. An icELISA was established based on a rabbit polyclonal antibody. The IC_50_ was 48 ng/mL, and the LOD was 1 ng/mL.

In this study, we synthesized a hapten (CPPU-COOH) via a one-step modification in the CPPU described in previous work [23] and produced a mAb with high sensitivity and specificity to the CPPU. BSA, OVA, KLH, and THY were used as the carrier proteins of artificial antigens. Based on this mAb, an icELISA for CPPU was characterized and optimized. The icELISA was applied in the determination of the CPPU levels in cucumber and orange samples, and its accuracy and reliability were validated by using LC-MS.

## 2. Materials and Methods

### 2.1. Reagents and Instrumentals

Reagents and instrumentals are presented in Supplementary Materials.

### 2.2. Synthesis of Hapten

**Figure 1:**
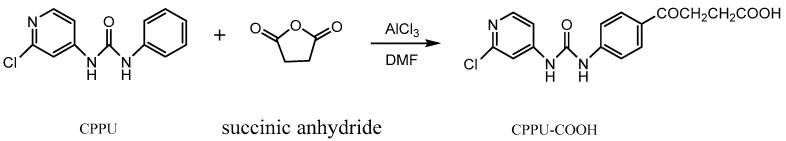
Synthesis route of the hapten. CPPU was coupled with succinic anhydride via Friedel–Crafts reaction, and a spacer arm was introduced on the phenyl ring of the CPPU.

The hapten CPPU-COOH was synthesized by coupling succinic anhydride with CPPU via a one-step Friedel–Crafts reaction as previously described [23]. Briefly, DMF (5 mL) was dropwise added to anhydrous AlCl_3_ (6.75 g) under stirring at 0 °C. After the AlCl_3_ was completely dissolved, CPPU (1.24 g) and succinic anhydride (0.5 g) were added to the reaction mixture and stirred at 50 °C for 6 h. Next, the reaction mixture was poured into ice water (60 mL) with the addition of concentrated HCl (4 mL). The precipitate was collected and washed with distilled water to yield a raw product. After recrystallization in DMF/methanol, the hapten CPPU-COOH was obtained and identified by LC-MS and ^1^H NMR (Figure 1).

### 2.3. Preparation of Artificial Antigens

Artificial antigens CPPU-COOH-OVA, CPPU-COOH-BSA, CPPU-COOH-KLH, and CPPU-COOH-THY were synthesized through active ester methods [24,25]. Briefly, CPPU-COOH (0.04 mmol), DCC (0.05 mmol), and NHS (0.05 mmol) were dissolved in DMF (600 μL) and kept stirring at 4 °C for 12 h. Then, the activated hapten was added to 5 mL of conjugate buffer (CBS) containing 50 mg of carrier proteins. Next, the conjugate mixture was dialyzed against PBS (10 mM, pH 7.4) at 4 °C for 72 h, and finally stored at −20 °C for further use.

### 2.4. Production of mAb

At the first immunization, each eight-week-old Balb/c female mouse was injected subcutaneously with 200 µL of an emulsion containing an equal volume of immunogen (1.0 mg/mL) and Freund’s complete adjuvant. In the subsequent booster immunizations, Freund’s incomplete adjuvant was substituted for Freund’s complete adjuvant and was injected at two-week intervals. After the third booster injection, the antisera were collected 7 days after each injection and checked by icELISA.

Hybridoma preparation procedures were performed as described previously [26]. The positive hybridoma cells were subcloned three to four times via the process of limiting dilution. The optimal antibody-generating clone was selected to generate ascetic antibodies. The anti-CPPU mAb was purified from ascites by the protein G affinity column method [27]. The subtype of the mAb was identified by using antibody isotyping kits.

### 2.5. Optimization of ELISA Conditions

The icELISA protocol was performed as described previously [28,29,30]. Briefly, 100 µL/well of coating antigen (1 µg/mL in CBS) was added to 96-well microtiter plates and stored at 37 °C overnight. The plate was rinsed twice with washing buffer and blocked by PBST containing 5% skimmed milk powder (120 µL/well) for 3 h at 37 °C. Then, the plate was rinsed twice and dried at 37 °C for 60 min. CPPU standard in PBS (50 µL) and the antibodies diluted in PBS (50 µL) were added and incubated at 37 °C for 40 min. After rinsing five times, the goat anti-mouse IgG-HRP was added (100 µL/well), followed by incubation for 30 min at 37 °C. After rinsing five times, the TMB solution (100 µL/well) was added and incubated for another 10 min at 37 °C. Stopping reagent (50 µL/well) was injected into each well to stop the chromogenic reaction, and the absorbance at 450 nm was measured using a microplate reader.

Many reports have shown that the immunoassay is an equilibrium binding reaction, and the experimental parameters used can affect the performance of the ELISA [28,31,32,33]. In order to improve sensitivity of the established icELISA, the assay was optimized on concentrations of coating antigen and antibody, working buffer, and IgG-HRP dilution multiple. A standard curve for CPPU was obtained under optimal conditions by four-parameter sigmoidal fitting with Origin 9.0 software (OriginLab Corporation, Northampton, MA, USA). The IC_10_, IC_20_, IC_50_, and IC_80_ values correspond to the CPPU concentration levels that inhibit 10%, 20%, 50%, and 80% of the binding between mAb and the coating antigen, respectively. The IC_10_ value represents the LOD of the assay. The concentration levels from IC_20_ to IC_80_ are the linear range of the assay [27,34,35,36].

### 2.6. Cross-Reactivity Test

To evaluate the specificity of the mAb against CPPU, CR values with six structural analogs were chosen for evaluation. The CR values were calculated using Equation (1) [26,37]:

CR (%) = IC_50 CPPU_/IC_50 structural analogue_ × 100 (1)

### 2.7. Detection of CPPU in Spiked and Real Samples

Cucumber and orange samples obtained from a local supermarket were detected as CPPU-negative by LC-MS analysis. The negative samples were spiked with series concentrations of CPPU for a recovery test. Eight blind samples were also chosen to be tested with both icELISA and LC-MS.

The samples were pretreated according to the Chinese National Standard method for CPPU detection in foods with some modifications [38]. A 10.00 g sample was mixed with 10.0 mL of acetonitrile and extracted for 3 min, then 4.0 g of anhydrous magnesium sulfate and 1.0 g of sodium chloride were added and shaken for 1 min followed by a centrifugation (4000 r/min, 5 min). An amount of 8.0 mL of the supernatants was mixed with 200 mg of primary secondary amine (PSA), 50 mg of graphitized carbon black (GCB), and 700 mg anhydrous magnesium sulfate, then extracted for 2 min, followed by a centrifugation (4000 r/min, 5 min). The final supernatants were filtered through 0.22 µm membrane before being analyzed by the established icELISA. The accuracy of the method was validated by LC-MS. Chromatographic separation was achieved in an Eclipse plus C_18_ column (2.1 × 50 mm, Agilent Technologies, Santa Clara, CA, USA) at a flow rate of 0.4 mL/min (mobile phase A was 10% methanol; mobile phase B was pure acetonitrile). The injection volume was 1 µL. The samples were quantified by ESI in the positive mode.

## 3. Results and Discussion

### 3.1. Hapten Synthesis and Artificial Antigen Preparation

CPPU is a small molecule (Mw < 1000 Da) without immunogenicity. Hapten design and synthesis are crucial processes in the development of specific antibodies. A complicated synthetic route may lead to decreasing yield and purity. In this study, one hapten (CPPU-COOH) was synthesized via a Friedel–Crafts reaction to introduce a four-carbon spacer arm at the phenyl ring of CPPU and identified by electrospray ionization mass spectrometry (ESI-MS) and NMR. As shown in the mass spectrum (Figure S1), the highest peak at m/z 349 was consistent with the mass-to-charge ratio of the parent ion of CPPU-COOH. The ^1^H NMR spectrum of CPPU-COOH (Figure S2) revealed the presence of two methylene groups (δ_H_ 3.20 (t, J = 6.3 Hz, 2H) and 2.56 (t, J = 6.3 Hz, 2H)), which correspond to two methylene groups from succinic anhydride. The proton signals at δ_H_ 7.96 (d, J = 8.7 Hz, 2H) and 7.61 (d, J = 8.8 Hz, 2H) can be attributed to these four protons at the phenyl ring of hapten CPPU-COOH. The proton signals at δ_H_ 8.20 (d, J = 5.7 Hz, 1H), 7.66 (d, J = 1.9 Hz, 1H), and 7.35 (dd, J = 5.7, 1.9 Hz, 1H) can be attributed to these three protons at the pyridyl ring of CPPU-COOH. The proton signals of the carboxyl group in CPPU-COOH are matched with δ_H_ 12.14 (s, 1H). The proton signals at δ_H_ 9.48 (s, 1H), and 9.40 (s, 1H) can be attributed to the two protons of ureido in CPPU-COOH. These results indicated the successful synthesis of CPPU-COOH.

**Figure 2:**
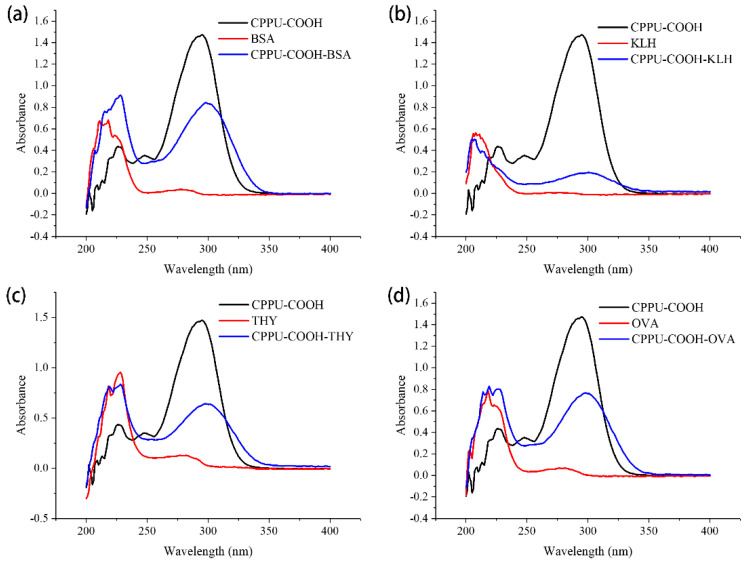
UV spectra of hapten, carrier proteins, and artificial antigens. (**a**) CPPU-COOH-BSA; (**b**) CPPU-COOH-KLH; (**c**) CPPU-COOH-THY; (**d**) CPPU-COOH-OVA. CPPU-COOH-BSA, CPPU-COOH-KLH, CPPU-COOH-THY, and CPPU-COOH-OVA exhibited a distinct pattern of absorbance, reflecting properties of the carboxyl on the hapten to amino groups of the carrier protein.

To produce a specific antibody, haptens should be coupled with proteins to form artificial antigens with both immunogenicity and immunoreactivity. BSA and OVA are commonly used proteins. In this study, BSA, KLH, and THY were selected as carriers and conjugated with CPPU hapten to obtain immunogens; BSA and OVA were chosen as substrates to obtain coating immunoreagents. The designed hapten CPPU-COOH contained a carboxyl group by which CPPU-COOH could be conjugated with amine groups in carrier proteins via the active ester method. The hapten–protein conjugates were characterized by UV spectrophotometry (Figure 2). As shown in Figure 2, CPPU-COOH has a characteristics absorption peak at 295 nm, whereas BSA, KLH, THY, and OVA displayed the characteristic absorption peak of proteins at ~280 nm. The obtained hapten–protein conjugates, CPPU-COOH-BSA, CPPU-COOH-KLH, CPPU-COOH-THY, and CPPU-COOH-OVA contained a distinct absorption peak at ~295 nm, which indicated that CPPU-COOH was successfully conjugated to the carrier protein [39].

### 3.2. Antisera and Coating Antigens Combination Selection

The titer and specificity of the antisera were assessed via icELISA. As shown in Table 1, antisera induced by immunogens CPPU-COOH-BSA and CPPU-COOH-KLH showed relatively low affinity to CPPU with lower inhibition rates (<55.9%), while antisera from immunogen CPPU-COOH-THY showed a significantly higher titer and inhibition rate than those of CPPU-COOH-BSA and CPPU-COOH-KLH. When using CPPU-COOH-BSA as a coating antigen, the inhibition rate was 83.3% to CPPU (1 μg/mL). As reported previously, THY was selected as a carrier protein of immunogen for its high immunogenicity [40,41,42]. CPPU-COOH-BSA and CPPU-COOH-THY were chosen for mAb production and the development of the icELISA.

### 3.3. Production of mAb and Establishment of icELISA

**Figure 3:**
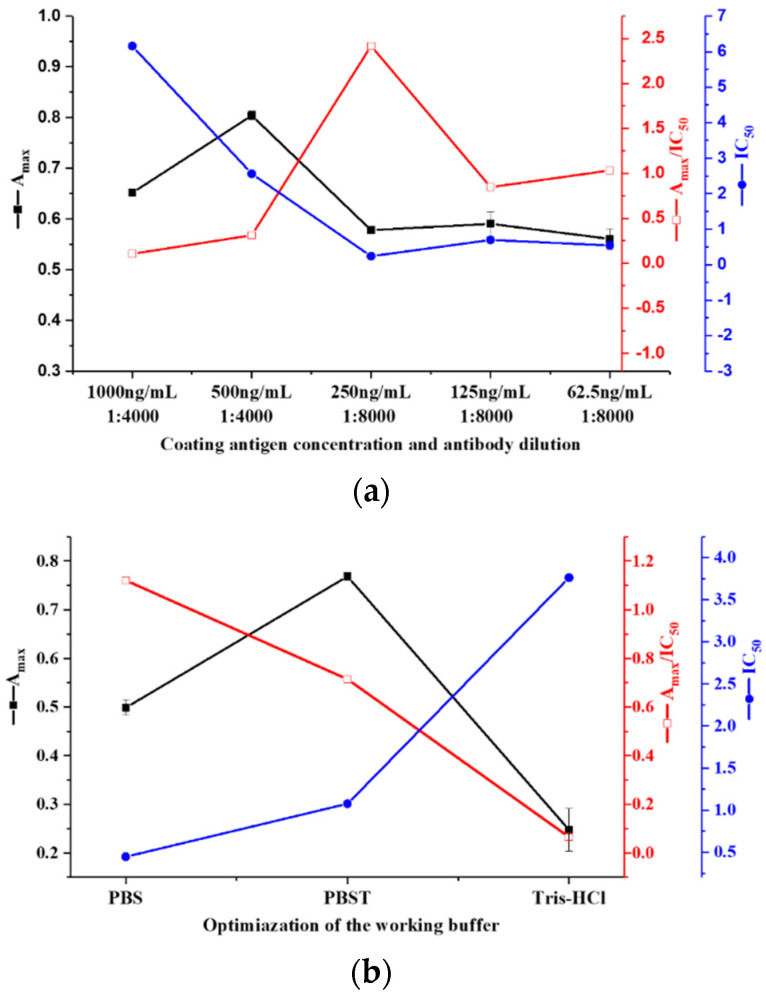

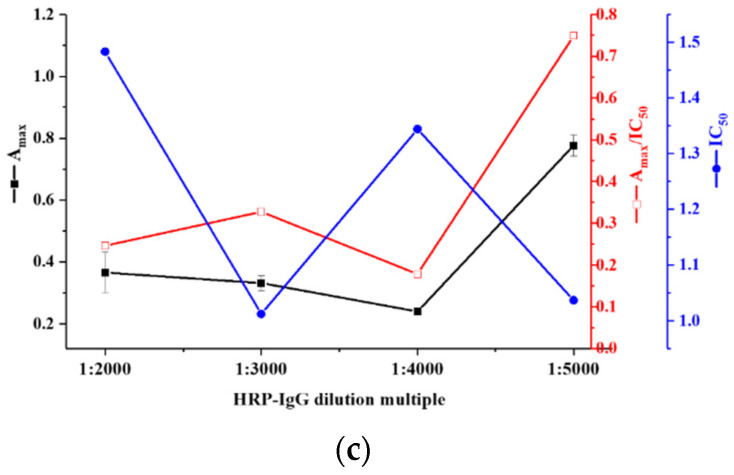
The optimized reaction conditions for icELISA (n = 3). (**a**) Concentration of coating antigen and antibody; (**b**) working buffer for the analyte; (**c**) IgG-HRP dilution multiple. The coating antigen at a concentration of 250 ng/mL, the antibody with a 1:8000 dilution (1 µg/mL), PBS as a dilution solution, and IgG-HRP diluted 1:5000 were chosen as the optimal working conditions.

**Figure 4:**
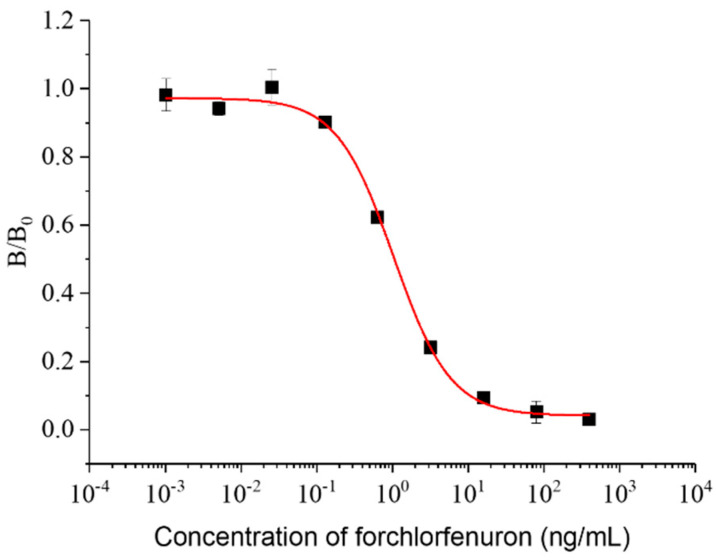
Standard curve for icELISA (n = 3). The IC_50_ for CPPU was 1.04 ng/mL, the LOD was 0.16 ng/mL, and the linear range was 0.31–3.43 ng/mL.

According to the results of the antisera detection, the mice immunized by CPPU-COOH-THY were selected for cell fusion. After four rounds of screening, the CPPU-specific mAb was produced from a selected hybridoma (CPPU-a). The subtype of the mAb was immunoglobulin G_1_ type. To enhance the assay sensitivity and stability, three parameters were optimized. The A_max_/IC_50_ ratio was used as the index for evaluating the influence of each condition; a higher ratio demonstrates a higher level of sensitivity in the assay [44,45,46,47]. The optimized results are shown in Figure 3. The A_max_ was the highest at 500 ng/mL of coating antigen and it decreased as the coating concentration became lower. Usually, a higher coating concentration can result in a higher A_max_ value, but an overly high coating concentration may cause mutual interference or increase non-specific adsorption when the immobilized coating antigen binds with the antibody, so the absorbance value may decrease after the blank value is subtracted. The IC_50_ gradually decreased as the content of the coating antigen and the antibody reduced and was the lowest at a coating concentration of 250 ng/mL and an antibody with 1:8000 dilution (1 µg/mL). The A_max_/IC_50_ ratio was also the highest at this concentration combination. So, these were chosen as the optimal working concentrations. The efficiency of icELISA can also be influenced by physicochemical factors related to the buffer [48]. The A_max_ and IC_50_ values varied when PBS, PBST, or Tris-HCl was used as a dilution solution of a standard analyte and antibodies. The A_max_/IC_50_ was more efficacious with PBS than with PBST or Tris-HCl with moderate A_max_ and the lowest IC_50_. This indicated that PBS is more suitable for the reaction system of this assay. The IgG-HRP was diluted from 1:2000 to 1:5000, and the highest A_max_ with the second lowest IC_50_ were observed at the dilution multiple of 1:5000, which displayed the highest A_max_/IC_50_ proportion. In an icELISA system, the content of the coating antigen, antibody, and secondary antibody (IgG-HRP) have a comprehensive effect on the sensitivity of the assay, so they should be optimized in accordance with each other. Under the optimum conditions, an icELISA standard curve was fitted. The IC_50_ value for CPPU was 1.04 ng/mL, the LOD was 0.16 ng/mL, and the linear range was between 0.31 and 3.43 ng/mL (Figure 4).

### 3.4. Cross-Reactions

The cross-reactivity (CR) is inversely related with the specificity. The CR was evaluated through IC_50_ of six CPPU structural analogues (Table 2). Low CR values were observed in the six listed compounds, which demonstrated that the mAb was specific and sensitive to CPPU. In a previous study [21], a CR with thidiazuron (TDZ) of 71% was observed, while in this study, the CR was 5.36% with TDZ and below 2% with the other analogues. This further confirmed the high specificity of the anti-CPPU mAb, which is vital to the established ELISA reaction system.

### 3.5. Detection of CPPU in Spiked and Real Samples

**Figure 5:**
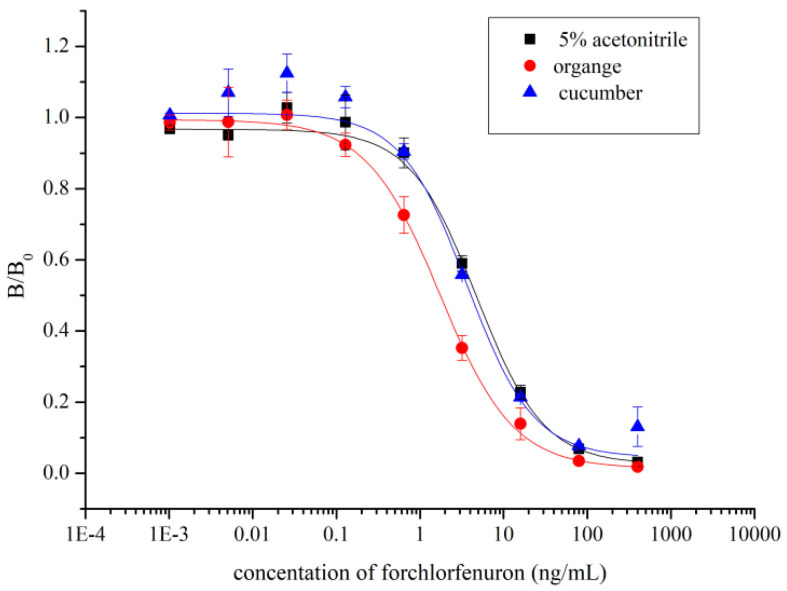
Matrix effects of cucumber and orange samples (n = 3). Standard curves were fitted using PBS containing 5% acetonitrile, spiked orange matrix (diluted with PBS containing 5% acetonitrile to twenty-fold), and spiked cucumber matrix (diluted with PBS containing 5% acetonitrile to twenty-fold). The three curves nearly overlap.

Generally, dilution of the sample can reduce the matrix effects on an immunoassay to a large extent [49,50]. In this study, spiked cucumber and orange samples were analyzed using icELISA. When diluted by PBS containing 5% acetonitrile to twenty-fold, the matrix effects were effectively eliminated (Figure 5).

Three different concentrations of CPPU were spiked in cucumber and orange samples and validated by icELISA. The average recovery rates of the samples were from 85.23% to 119.14% (Table 3), and the coefficients of variation (CV) were less than 10%.

Eight real samples were selected for detection with icELISA and the results were confirmed by LC-MS. As shown in Table 4, cucumbers number 1 and 3 and orange number 4 were detected positive by the established icELISA method. Good correlations were observed between icELISA and LC-MS, proving the high accuracy and reliability of the established icELISA method for CPPU quantitative determination in food samples.

## 4. Conclusions

In this study, a high-affinity and specific mAb against CPPU was produced and an icELISA was established for reliable CPPU residue analysis in food. The spiked and real food samples were analyzed with the icELISA, and the accuracy of the method was confirmed by LC-MS.

A hapten synthesis route with more simplicity and efficiency than previously reported methods [19,20,21,22] was utilized. With the mAb generated and the ELISA developed, CPPU was able to be detected at 0.16 ng/mL (3.2 μg/kg in food). The IC_50_ (1.04 ng/mL or 4.2 nM) was higher than that of the Abad-Fuentes group (IC_50_ < 1 nM); however, they obtained a limit of quantification of 5 μg/kg for kiwifruit, slightly higher than in this assay. They also reported a CR of 71% with the herbicide TDZ [21], while with this method, the CRs with TDZ and five other CPPU structural analogues were all less than 6%. Xiao [23] coupled CPPU with succinic anhydride via a one-step Friedel–Crafts reaction for the first time. IcELISA was developed by using a rabbit polyclonal antibody. The IC_50_ (48 ng/mL) and LOD (1 ng/mL) were both far higher than in this assay. In this study, we designed the synthesis route of hapten while referring to the synthetic method of Xiao. BSA (for immunogen) paired with OVA (for coating) were selected as carriers in previous studies [19,20,21,22,23]. In our work, KLH and THY were also selected as carriers for obtaining immunogens and compared to BSA. We found that antisera from THY immunogen showed significantly higher titer and inhibition rates than those from BSA and KLH, which indicated that carrier selection may be an effective way to improve CPPU antibody performance. A monoclonal antibody-based immunoassay is ideal for CPPU monitoring, due to its satisfactory accuracy, rapidity, and cost-effectiveness. Furthermore, this work aims to promote the application of more IAs in CPPU and in the detection of other agrochemicals.

## Figures and Tables

**Table 1:** Characterization of mice antisera against CPPU with different coating antigens.

Immunogen	CPPU-COOH-BSA		CPPU-COOH-KLH		CPPU-COOH-THY	
Coating Antigens	Titer ^a^ (10^3^)	Inhibition ^b^ of CPPU (%)	Titer ^a^ (10^3^)	Inhibition ^b^ of CPPU (%)	Titer ^a^ (10^3^)	Inhibition ^b^ of CPPU (%)
CPPU-COOH-BSA	-	-	16	55.6	32	83.3
CPPU-COOH-OVA	32	58.4	8	55.9	16	82.9

^a^ Titer means the dilution factor of antiserum with an absorbance of 450 nm at about 1.0–1.5 [25,43]. ^b^ Inhibition = [(B − B_0_)/B] × 100%, at 1 μg/mL of competitor [25,43].

**Table 2:** Cross-reactivity of CPPU and other structural analogues.

Analyte	Structure	IC_50_ (ng/mL)	Cross-Reactivity (%)
Forchlorfenuron (CPPU)	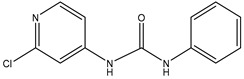	1.04	100
3,6-Dichloropicolinic	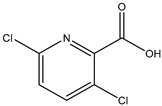	166.20	0.63
Linuron	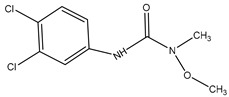	226.43	0.46
Thidiazuron (TDZ)	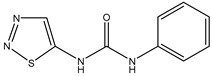	19.42	5.36
Picloram	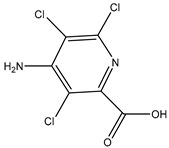	450.72	0.23
Chlortoluron	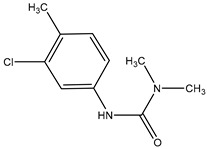	64.34	1.62
Diuron	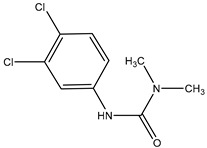	823.79	0.13

**Table 3:** Recoveries of CPPU-spiked food samples determined by icELISA (n = 3).

Samples	Spiked (ng/g)	Measured ± SD (ng/g)	Recovery (%)	CV (%)
Cucumber	40	38.70 ± 0.16	96.80	8.33
	80	79.00 ± 0.25	98.80	6.25
	160	159.20 ± 0.40	99.60	5.01
Orange	40	47.60 ± 0.14	119.14	6.21
	80	70.20 ± 0.11	85.23	3.11
	120	104.20 ± 0.36	86.88	6.82

**Table 4:** Comparison between icELISA and LC-MS quantitative results for CPPU residues in cucumbers and oranges.

Sample	Number	icELISA		LC-MS	
		ng/mL	ng/g	ng/mL	ng/g
Cucumber	1	5.12	102.4	5.17	103.4
	2	ND	ND	0.13	2.6
	3	1.96	39.2	2.62	52.4
	4	ND	ND	0.12	2.4
Orange	1	ND	ND	0.14	2.8
	2	ND	ND	0.06	1.2
	3	ND	ND	0.13	2.6
	4	0.32	6.4	0.35	7.0

ND: Not detected.

## Data Availability

Not applicable.

## Supplementary Materials

The following supporting information can be downloaded at: https://www.mdpi.com/article/10.3390/bios12020078/s1, Reagents and Instrumentals; Figure S1: The MS spectrum of CPPU-COOH; Figure S2: The NMR spectrum of CPPU-COOH.
