# Supplementary material for: A Sensitive Monoclonal-Antibody-Based ELISA for Forchlorfenuron Residue Analysis in Food Samples
Source: Biosensors (Basel). 2022 Jan 28;12(2):78. doi: 10.3390/bios12020078 (PMC8869720; doi:10.3390/bios12020078)
Supplement: Supplementary file 1 [file biosensors-12-00078-s001.zip › biosensors-1502975-supplementary.pdf]

biosensors

MDPI

Supplementary Materials: **A Sensitive Monoclonal-Antibody-Based ELISA for Forchlorfenuron Residue Analysis in Food Samples**

Xinmei Liu, Bo Xie, Yongjian Cheng, Lin Luo, Yifan Liang and Zhili Xiao *

Reagents and Instrumentals

Forchlorfenuron (CPPU) and succinic anhydride were purchased from Heowns Biochem Technologies Co., Ltd. (Tianjin, China). Aluminum chloride was purchased from Shanghai Macklin Biochemical Co., Ltd. (Shanghai, China). Freund's complete and incomplete adjuvants, ovalbumin (OVA), bovine serum albumin (BSA), keyhole limpet hemocyanin (KLH), thyroglobulin (THY), N,N'-dicyclohexylcarbodiimide (DCC), N-hydroxy succinimide (NHS), Polyethylene glycol (PEG) 4000,and antibody isotyping kits were obtained from Sigma Company (Ronkonkoma, NY, USA). 3',3,5,5'-tetramethylbenzidine (TMB) was obtained from Beijing Solarbio Science & Technology Co., Ltd. (Beijing, China). Horseradish peroxidase-labeled goat anti-mouse IgG (IgG-HRP) was purchased from TransGen Biotech (Beijing, China). N,N-dimethylformamide (DMF) was obtained from Damao Chemical Reagent Co., Ltd. (Tianjin, China). RPMI 1640 medium, hypoxanthine–aminopterin–thymidine (HAT), hypoxanthine–thymidine (HT), and glutamine (Gln) were purchased from Thermo Fisher Scientific Inc. (Waltham, MA, USA). Fetal bovine serum (FBS) was obtained from ExCell Bio Company (Taicang, China). Solutions for ELISA were prepared as follows: (1) CBS: 50 mmol/L carbonate buffer (pH 9.6) for coating; (2) PBS: 10 mmol/L phosphate buffer (pH 7.4) for working buffer; (3) PBST: PBS with 0.05% Tween-20 for washing and general diluent; (4) 5% skimmed milk powder in PBST for blocking; (5) 2 mol/L H_2SO_4 as the stopping regent.

A Multiskan MK2 microplate washer (Thermo Fisher Scientific, Waltham, MA, USA) was used to wash the ELISA plates. Absorbance was detected at a wavelength of 450 nm using a Multiskan MK3 microplate reader (Thermo Fisher Scientific, Waltham, MA, USA). Ultraviolet spectrometry (UV) was recorded on a UV-3010 spectrophotometer (Hitachi, Tokyo, Japan). Nuclear magnetic resonance (NMR) spectra were recorded with either a DRX-400 or DEX-600 NMR spectrometer (Bruker, Rheinstetten, Germany). Liquid chromatography tandem mass spectrometry (LC-MS) was carried out using a 1200 series LC system (Agilent Technologies, Santa Clara, CA, USA).

The SP2/0 myeloma cell line was stored in our laboratory. Female Balb/c mice were supplied by Guangdong Medical Experimental Animal Center (Foshan, China). All the animal experiments were performed in compliance with the protective and administrant laws for laboratory animals of China, and conducted with the approval of the Institutional Authority for Laboratory Animal Care.

Cucumber and orange samples were purchased from local supermarkets.

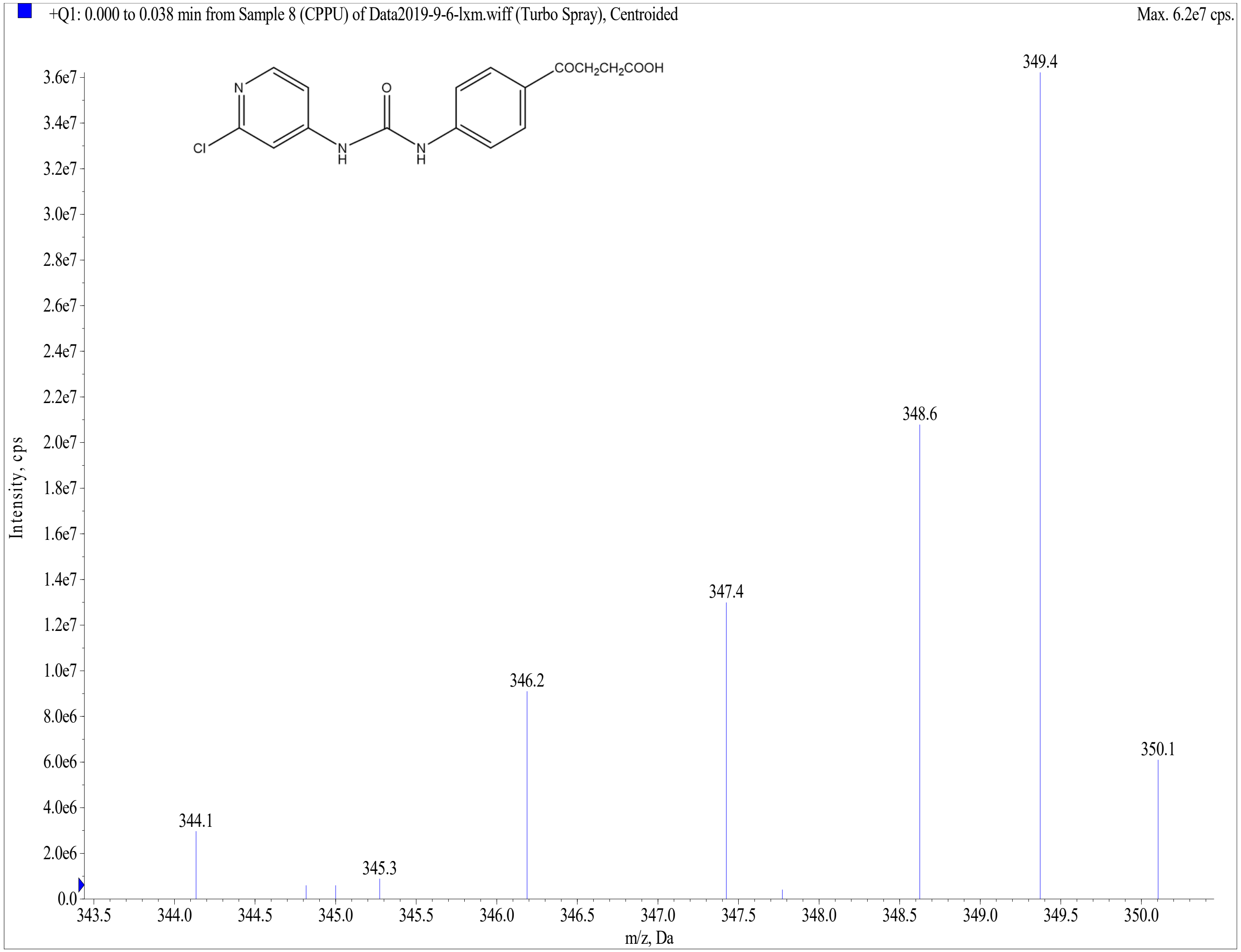

Figure S1. The MS spectrum of CPPU-COOH.

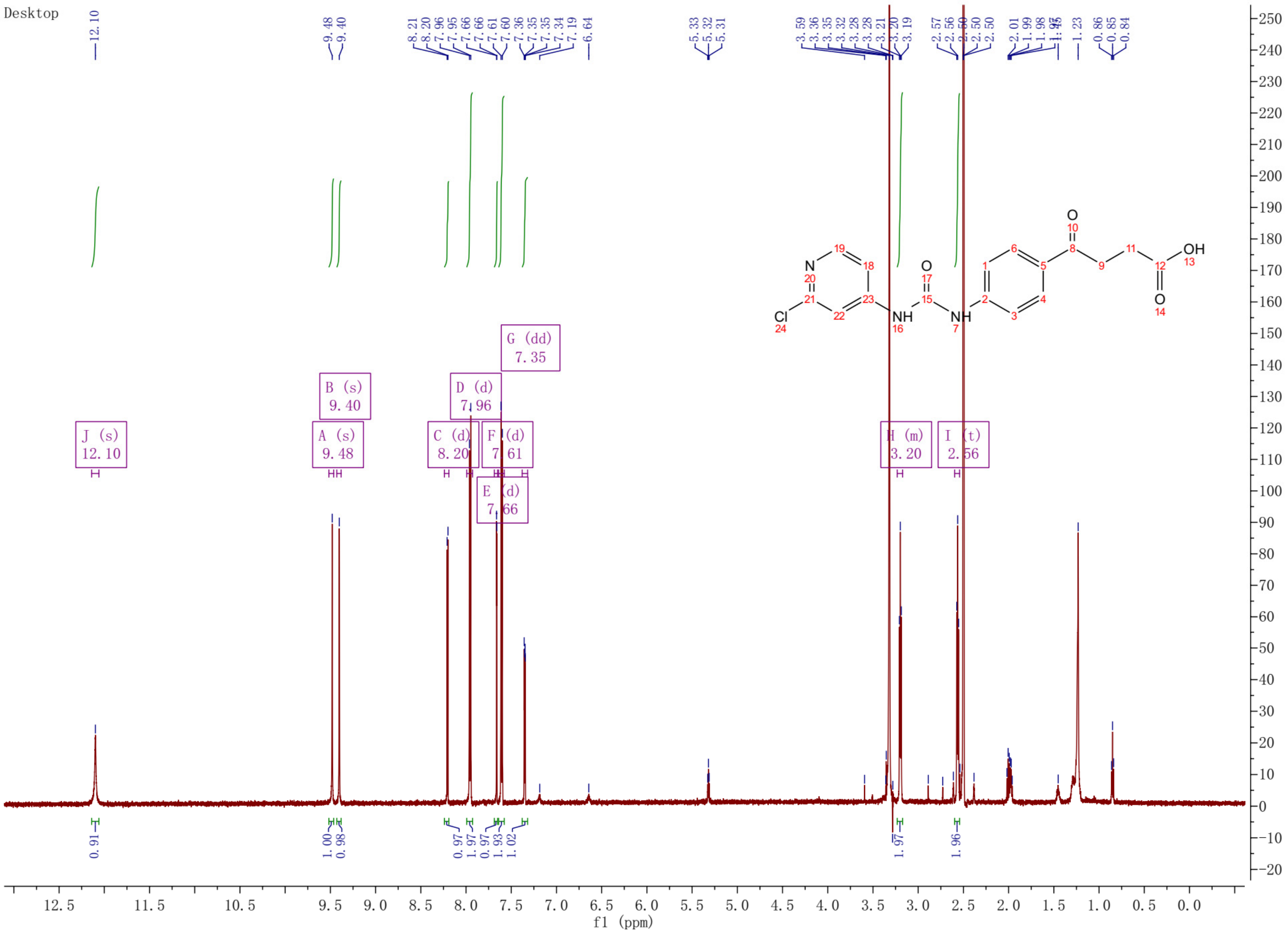

Figure S2. The NMR spectrum of CPPU-COOH.
